# Performance Evaluation of Manual and Automated (MagNA Pure) Nucleic Acid Isolation in HPV Detection and Genotyping Using Roche Linear Array HPV Test

**DOI:** 10.1155/2011/931281

**Published:** 2011-07-09

**Authors:** Aikaterini Chranioti, Evangelia Aga, Niki Margari, Christine Kottaridi, Asimakis Pappas, Ioannis Panayiotides, Petros Karakitsos

**Affiliations:** ^1^Department of Diagnostic Cytopathology, University General Hospital “Attikon”, Rimini 1, Chaidari, 12462 Athens, Greece; ^2^3rd Department of Obstetrics and Gynecology, University General Hospital “Attikon”, Rimini 1, Chaidari, 12462 Athens, Greece; ^3^2nd Department of Pathology, University General Hospital “Attikon”, Rimini 1, Chaidari, 12462 Athens, Greece

## Abstract

Nucleic acids of human papillomavirus (HPV) isolated by manual extraction method (AmpliLute) and automated MagNA pure system were compared and evaluated with cytohistological findings in 253 women. The concordance level between AmpliLute and MagNA was very good 93.3% (*κ* = 0.864, *P* < .0001). Overall HPVpositivity detected by AmpliLute was 57.3% (30.4% as single and 27% as multiple infections) in contrast to MagNA 54.5% (32% and 23%, resp.). Discrepant results observed in 25 cases: 11 MagNA(−)/AmpliLute(+), 10 of which had positive histology; 5 MagNA(+)/AmpliLute(−) with negative histology; 8 MagNA(+)/AmpliLute(+): in 7 of which AmpliLute detected extra HPV genotypes and 1 MagNA(invalid)/AmpliLute(+) with positive histology. Both methods performed well when compared against cytological (area under curve (AUC) of AmpliLute 0.712 versus 0.672 of MagNA) and histological diagnoses (AUC of AmpliLute 0.935 versus 0.877 of MagNA), with AmpliLute showing a slightly predominance over MagNA. However, higher sensitivities, specificities, and positive/negative predictive values were obtained by AmpliLute.

## 1. Introduction

It is now well established and widely accepted that virtually all cervical cancer and its immediate precancerous lesions arise from persisting cervical infection by some highly oncogenic HPV genotypes [1, 2]. The most important of these HPV genotypes are HPV16 and HPV18 which account for ~70% of all invasive cervical cancers with minor variations in this percentage between continents [3].

Fifteen HPV genotypes have been to date classified as high-risk (HR) types (16, 18, 31, 33, 35, 39, 45, 51, 52, 56, 58, 59, 68, 73, and 82), 3 as probably HR (26, 53, 66) and 12 as low-risk (LR) (6, 11, 40, 42, 43, 44, 54, 61, 70, 72, 81, and CP6108) [1, 4].

The majority of HPV infections are transient, but persistence of an HR HPV is a significant risk factor for the development of cervical cancer. This occurs only in a minority of infections and is an unpredicted event. It could be a genetic predisposition with an inadequate immune response and/or possible uncontrolled reaction with tumor suppressor genes [5, 6]. 

Type-specific detection of HPV is increasingly important for monitoring the impact of HPV vaccine implementation and as a tool for cervical cancer screening. As a consequence, standardization of laboratory methods for HPV detection and typing is important. The commercial HPV detection kits: Hybrid Capture II (Digene Corporation, Gaithersburg, Md, USA), Cervista HPV HR (Third Wave Technologies, Inc., Madison, USA) and Cervista 16/18 tests are approved by the FDA for use in routine screening of HPV. However, the above assays are unable to discriminate specific genotypes or to identify infections involving multiple genotypes and the Cervista assay detects only two HPV types (types 16 and 18).

Various molecular assays for HPV detection and typing have been used in epidemiological studies, and they are based on two different technologies: (1) hybridization-based assays (e.g., HC II) and (2) PCR-based tests (e.g., GP5+/GP6+, PGMY09/11, INNO-LiPA HPV Genotyping (Innogenetics, Belgium), Linear Arrays HPV test (Roche Molecular Systems, Inc. Branchburg, NJ, USA), CLART HPV2 (Genomica, Madrid, Spain). The advantages and disadvantages of these two basically different methodologies have been extensively discussed [7–19]. 

The qualitative Linear Array HPV (LA-HPV) HPV genotyping test, developed by Roche Molecular Systems offers a reliable, sensitive, and standardized approach for HPV typing in cervical specimens. It is distributed as a research use only but it has been submitted for FDA review. This test utilizes amplification of target DNA by PCR and nucleic acid hybridization for the detection of 37 types in cervical cells collected into an LBC media. This test includes four steps: specimen preparation—DNA extraction, PCR amplification, hybridization of the amplified products with specific probes and colourimetric detection on the hybrids on strip [13, 17, 20–22]. Current specimen processing protocols recommend the use of manual extraction of DNA using the AmpliLute liquid media extraction kit, based on the QIAamp method (QIAGEN, Inc., Valencia, Calif, USA). An alternative method for DNA extraction is the automated MagNA Pure LC extraction system, developed by the same company.

The objective of this study was to evaluate and compare the automated MagNA pure DNA extraction method with the AmpliLute DNA extraction method in detecting HPV DNA form ThinPrep Pap tests using the linear array (LA) HPV genotyping and detection assays and also to correlate these results to cytological and histological diagnosis.

## 2. Methods

### 2.1. Clinical Specimens

In the present study, cervical brush specimens were obtained from women aged from 17 to 70 years who attended the gynecologic outpatient clinic of “Attikon” University Hospital, Athens, Greece, for opportunistic examination, between July 2009 and May 2010. Women considered eligible for the study if they fulfilled the following criteria: (a) they agreed to undergo colposcopy and if necessary cervical biopsy and (b) there was enough residual biological material, after cytological examination, for the two molecular assays to be completed. A total of 253 women met these criteria and were enrolled in the study. This patient population does not represent the general population of women attending public screening programs. Approval from the ethics committee was obtained before inclusion.

### 2.2. Cytological Diagnosis

Samples of ThinPrep Pap tests were collected by means of a Brun's-like brush. The PreservCyt vials (Corporate Headquarters: Hologic, Inc., Ltd., UK), containing the cell samples were addressed to the Department of Cytopathology of the aforementioned hospital for preparation of thin-layer slides using the ThinPrep 2000 Automated Slide Processor (Corporate Headquarters: Hologic, Inc., Ltd., UK) according to the manufacturer's instructions. Cytological findings were interpreted according to the Bethesda classification system and were classified as follows: (a) within Normal Limits (WNL), (b) atypical squamous cells of undetermined significance (ASC-US), (c) low-grade squamous intraepithelial lesion (LSIL), and (d) high-grade squamous intraepithelial lesion (HSIL). The cytopathologists and the biologist conducting HPV testing were all blinded to the clinical profile to ensure unbiased reporting.

### 2.3. Histological Diagnosis

A cervical biopsy was performed if lesions were present upon colposcopy. All histological assessments were made blinded to the HPV DNA status of the participants. The histological evaluation revealed the following categories: negative HPV, CIN 1, CIN 2, and CIN 3. In case histology showed a CIN 2 or CIN 3, the patient was referred for appropriate treatment.

### 2.4. DNA Extraction Methods

After slide preparation for cytological examination, the remaining PreservCyt samples were vortexed vigorously for 15 sec to maximize homogeneity and two aliquots of 250 *μ*L and 1 mL were generated from each clinical specimen.

DNA was isolated using two different procedures (i) a 250 *μ*L aliquot was extracted by AmpliLute liquid media kit (Roche Molecular Systems) in conjunction with a QIAvac 24 plus vacuum system, according to the manufacturer's instructions in the product insert and (ii) a 1 mL aliquot was extracted by MagNA Pure LC extraction system using the DNA-I kit (blood cells high-performance protocol) (Roche Molecular Systems). Briefly, for the manual extraction, samples and HPV positive/negative controls were processed in parallel. Clinical samples were mixed by vortexing to form a homologous state, and 250 *μ*L were removed and lysed with proteinase K solution and buffer ATL at 56°C for 30 min. The samples underwent a second incubation at 70°C for 15 min in the presence of buffer AL containing a carrier RNA. The lysate was then transferred to vacuum columns where isolation and purification of DNA was completed via washing of different solutions to bind DNA and remove other cellular materials. Extracted DNA was eluted into 120 *μ*L of buffer AVE. Specimens and controls were immediately stored at 2°C–8°C for up to 7 days or frozen at −20°C for up to 8 weeks.

For automated extraction, the samples were prepared using a modified procedure involving the centrifugation of 1 mL aliquots of the PreservCyt samples at 13000 × g for 20 min prior to discarding of the supernatant. The resulted cell pellets were resuspended into 200 *μ*L of sterile phosphate-buffered saline, and the procedure of automated extraction was followed according to the manufacturer's instruction, using the DNA-I kit. The method is based on magnetic-bead technology with a special buffer containing chaotropic salts and proteinase K. Nucleic acids are bound to the surface of the magnetic glass particles. Cellular debris was removed by several washing steps, and the purified nucleic acids were eluted. 100 *μ*L in volume of extracted genomic DNA product was obtained, after the magnetic beads were separated from the solution. Specimens and controls were immediately stored at 2°C–8°C for up to 7 days or frozen at −20°C for up to 8 weeks. After nucleic acid purification all samples were analyzed by LA HPV assay for HPV genotyping.

### 2.5. LA-HPV Amplification (PCR)

The LA genotyping test use a pool of biotinylated primers designed to amplify an approximately 450 bp sequence within the polymorphic L1 region of the genome of the 37 HPV genotypes. An additional 268 bp primer pair which targets the human *β*-globin gene is included in the assay to provide a control for cell adequacy, extraction, and amplification. PCR was carried out on each of the samples and controls, using the Linear Array HPV genotyping mastermix which contains: Tris buffer, potassium chloride, AmpliTaq, gold DNA polymerase (microbial), AmpErase, (uracil-N-glycosylate) enzyme (microbial), dATP, dCTP, dUTP, dGTP, dTTP, each of upstream and downstream primers (biotinylated) and *β*-globin primers, sodium azide, magnesium chloride, and amaranth dye. The reaction mixture contained 50 *μ*L of HPV mastermix and 50 *μ*L of eluted DNA. The amplification was performed on the Applied Biosystems Gold-plated 96-well GeneAmp PCR System 9700 (Applied Biosystems, Foster City, Calif, USA) using the following thermal profile: 2 min at 50°C, 9 min at 95°C; 30 sec at 95°C, 1 min at 55°C, 1 min at 72°C (40 cycles) with a ramp rate set at 50% followed by 5 min at 72°C and a final hold at 72°C indefinitely. PCR amplicons were immediately denatured by the addition of 100 *μ*L of (DN) denaturation reagent and stored at 4°C for further analysis within 7 days.

### 2.6. LA-HPV Detection

The detection of the HPV genotypes was carried out using the LA HPV detection kit. Once the amplification was completed, 75 *μ*L of denatured amplicon were added to the linear array strips that contain multiple copies of HPV genotype-specific probes in a defined area for all 37 genotypes and the *β*-globin reference lines. The HPV types detected are HPV6, 11, 16, 18, 26, 31, 33, 35, 39, 40, 42, 45, 51–56, 58, 59, 61, 62, 64, 66–73, 81–84, IS39, and CP6108. The biotin-labeled amplicon was bound to the strips using a hybridization buffer in a shaking waterbath at 53°C. Once bound, the strips were washed at high stringency to remove nonbound material and streptavidin-horseradish peroxidase conjugate was then added and bound to the biotin-labeled amplicon hybridized to the oligonucleotide probes. The strips were then washed with a substrate solution containing hydrogen peroxide and 3,3′,5,5′-tetramethylbenzidine (TMB). In the presence of hydrogen peroxide, the bound streptavidin-horseradish peroxidase catalyzed the oxidation of TMB to form a blue-colored complex, which precipitated at the probe positions where hybridization had occurred (colourimetric determination). This color precipitation allowed for manual reading of the strips and genotype detection by comparison with the HPV reference guide provided. LA test does not directly detect HPV52 but combines a set of probes that detects HPV33, −35 and −58 (HPV mix). Specimens that test negative for HPV33, −35 and −58 individually but are positive for HPV mix are considered to be HPV52 positive. The specimens that test positive for HPV mix and for HPV33, −35 and/or −58 have an uncertain HPV52 status, for this analysis, these specimens were considered to be HPV52 negative, since coinfection with HPV52 cannot be ruled out by this test.

The procedure performed into two physically separated areas (pre-PCR and post-PCR) in order to avoid contamination of samples with previously amplified products. All washes and hybridization steps were undertaken in a 24-well tray with lid. The reading of the strips, produced by the two methods, was made by one well-experienced biomedical scientist. Discrepant interpretations were resolved by a second biomedical scientist and consensus review performed without knowledge of prior results. The LA-HPV test does not cross-react with a variety of viruses, bacteria, protozoa and yeast that could be present in cervical specimens.

### 2.7. Statistical Analysis

Pairwise comparison of AmpliLute method and MagNA pure method was performed by using kappa (*κ*) statistics. A *κ* value of 0 indicates no agreement better than chance, and *κ* value of 1 indicates perfect agreement, *κ* values from 0 to 0.20, 0.21 to 0.40, 0.41 to 0.60, 0.61 to 0.80 and 0.81 to 1.00 indicate poor, fair, moderate, good, and very good strengths of agreement, respectively. All *P* values <.001 are considered statistically significant. Receiver operating characteristics (ROC) curve analysis was applied to calculate and compare AmpliLute method and MagNA pure method with cytological findings. In addition ROC analysis was applied to calculate and compare AmpliLute method and MagNA pure method with histological findings. All the statistical analyses were obtained with the statistical package for the social sciences (SPSS) computer software.

## 3. Results

A total of 253 women were analyzed in the present study by means of screening for the presence of HPV DNA by using two different DNA extraction methods. Out of the 253 cervical smears, 253 nucleic acid extracts were produced using the AmpliLute liquid media extraction method and 253 DNA extracts were generated using the MagNA pure automated extraction system. All women referred to colposcopy and if visible lesions were found, they were sampled. Patients with severe cervical diseases were further assessed.

The DNA extracts were evaluated by the LA-HPV genotyping test and compared against the reported cytological and histological diagnoses. The levels of sample adequacy for cytological examination and for nucleic acid extraction and amplification efficiency among the specimens, based on *β*-globin positivity, did not differ dramatically between the tests (Table 1). Sample adequacy was higher with AmpliLute extracts (100%) than with the MagNA pure method (99.6%) and cytology examination (97.6%). Only one nucleic acid extract generated by the MagNA pure LC was invalid after LA genotyping test due to the absence of high and low *β*-globin result. 

The comparison of HPV LA test results using AmpliLute extracts with equivalent MagNA pure extracts showed an overall concordance of 93.3% (*κ* = 0.864, *P* < .001) (Table 2). HPV genotype profiles were identical in 228/253 (90.1%) of specimens, including of 103 HPV negative samples and 125 positive samples with identical HPV status detected by both methods. Out of 125 cases, the same HPV profile was identified in 73 single infections and in 52 multiple infections. Discrepant results observed in 25 samples including 11 cases which were AmpliLute(+)/MagNA(−), 5 cases AmpliLute(−)/MagNA(+), 5 cases identified as single infections in MagNA but as multiple type infections in AmpliLute, 3 cases in which multiple infections were detected with different HPV profile between the two methods, and in one case, MagNA generated an invalid result as opposed to AmpliLute (Table 3). 

Overall, 94/253 (37%) women were diagnosed with normal cytology, 50/253 (19.8%) were exhibited ASCUS, 91/253 (36%) were diagnosed with LSIL, 5/253 (2%) with LSIL but having some positions with HSIL, and 7/253 (3%) with HSIL. In 6 cases (2.4%) the cytological diagnosis was difficult due to inadequacy of the clinical samples (Tables 4 and 5).

HPV positivity detected by AmpliLute was slightly higher compared with MagNA Pure, 57.3% and 54.9%, respectively. The largest percentage of samples negative for HPV DNA was found in WNL category. Analytically, for the cytological category WNL, HPV positivity observed by AmpliLute method was 29/94 (31%), for ASCUS was 46%, for LSIL 77%, for LSIL/HSIL 100% and for HSIL 100%. HPV positivity detected by the MagNA pure LC method for the aforementioned categories was: 33%, 44%, 78%, 80%, and 100%, respectively, (Tables 4 and 5).

**Figure 1:**
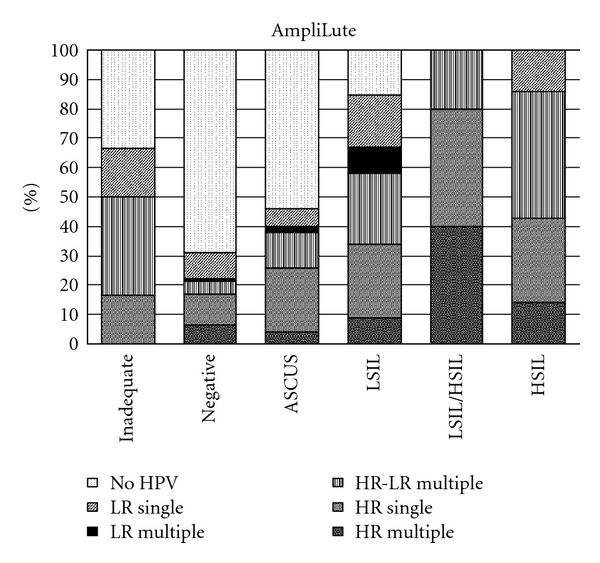
Prevalence of HR-HPV and LR-HPV types according to cytological diagnosis as detected by the AmpliLute extraction method.

**Figure 2:**
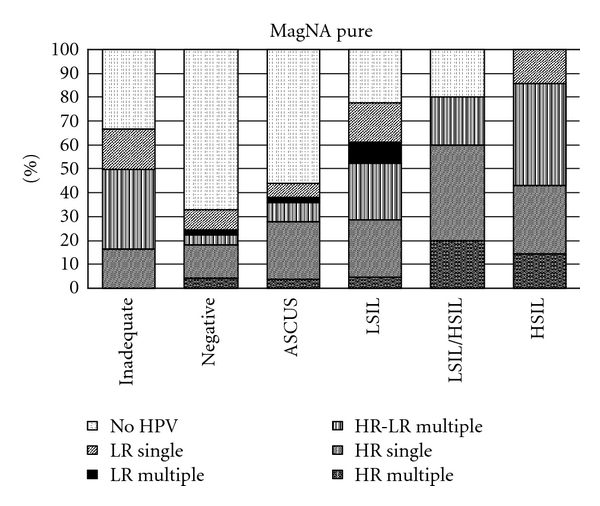
Prevalence of HR-HPV and LR-HPV types according to cytological diagnosis as detected by the MagNA pure extraction method.

Within the studied population, single and multiple type infections were present in every cytological diagnosis. In total, single HPV infections detected by the AmpliLute was 28.5% and by MagNA Pure 30.4% whereas multiple type of infection was observed in 26.5% and 23%, respectively. In this study, multiple infections composed of up to five HPV genotypes in a plethora of combinations. The HPV distribution of single infections (divided into two categories according to the HPV genotype oncogenicity: LR and HR) as well as multiple infections (divided into three categories: LR (when only low-risk HPV types were present), HR-LR (when low- and high-risk HPV types were present) and HR (when only high-risk HPV types were present) in all cytological categories studied are given in Figure 1 for the AmpliLute method and in Figure 2 for the MagNA pure. More multiple infections were detected by AmpliLute method in all cytological categories compared with the MagNA pure (*P* not statistical significant). Analytically, for the cytological category WNL, the composition of multiple infections observed by AmpliLute method was at 6.4% with HR, 4.2% with HR-LR, and 1% with LR, for ASCUS at 4% with HR, 12% with HR-LR, and 2% with LR, for LSIL at 9% with HR, 24% with HR-LR, and 9% with LR, for LSIL/HSIL at 40% with HR and 20% with HR-LR and for HSIL at 40% with HR, 20% with HR-LR. Multiple infections detected by the MagNA Pure LC method for the aforementioned categories was WNL at 4.2% with HR, 4.2% with HR-LR, and 2% with LR, for ASCUS at 4% with HR, 8% with HR-LR and 2% with LR, for LSIL at 4% with HR, 23% with HR-LR and 9% with LR, for LSIL/HSIL at 20% with HR and 20% with HR-LR and for HSIL at 14% with HR, 43% with HR-LR. The HPV type prevalence according to the two extraction methods is given below with HPV16 being the most frequent type detected, in both types of infections and by the two methods, followed by HPV31, HPV53, HPV6, HPV33, HPV45, HPV42, and HPV51 as detected by the AmpliLute method and by HPV31, HPV53, HPV18, HPV51, HPV18, HPV6, and HPV33 as detected by the MagNA method (data not showed).

 The two extraction methods were compared against the cytological findings (inadequate cytological samples were excluded). In terms of processing evaluation, AmpliLute method obtained better results than MagNA pure method (AmpliLute: sensitivity (SE) = 73.2%, specificity (SP) = 69.15%, positive predictive value (PPV) = 79.43%, negative predictive value (NPV) = 61.32%; MagNA Pure: SE = 67.32%, SP = 67.02%, PPV = 76.87%, NPV = 55.75%). Both methods performed well when compared against the cytological diagnosis; nevertheless, the AmpliLute method demonstrated a slightest higher area under curve (AUC) 0.712 (Std. Error 0.035, 95% CI: 0.644–0.779, *P* < .001) compared to AUC of MagNA pure method 0.672 (std. error 0.036, 95% CI: 0.602–0.742, *P* < .001). 

The comparison of ThinPrep diagnosis and histological results is presented at Table 6. Out of 253 colposcopic examinations, 145 women underwent biopsy. In 108 women, no visible lesions were found, and therefore, those women were not sampled. Positive histological result was found in 129 cases. From patients with normal cytology, only 13 out of 94 (13.8%) in biopsy had a HPV lesion or CIN 1 diagnosis. From patients with ASCUS, 14 out of 50 (28%) had HPV in biopsy, 8/50 (16%) had CIN 1, whereas only 1 (2%) patient had CIN 2. In LSIL, 29/91 (31.8%) had a biopsy diagnosis of HPV, 39/91 (42.8%) had CIN 1, 7/91 (7.7%) had CIN 2, and only 2/91 (2.2%) had CIN 3. All cases classified as HSIL with ThinPrep cytology had a biopsy diagnosis of either CIN2 (4/7, 57.2%) or CIN3 (3/7, 42.8%). In LSIL/HSIL category, 1 patient (20%) had CIN1, 3/5 (60%) had CIN2 and 1/5 (20%) had CIN3. Four patients with no cytological diagnosis, due to inadequate of sampling, biopsy revealed lesions with HPV. 

Results of biopsy reading and HPV genotyping by the two extraction methods are demonstrated in Tables 7 and 8. Once more, AmpliLute method showed greater performance over MagNA pure (AmpliLute: SE = 100%, SP = 87.5%, PPV = 98.47%, NPV = 100%, FPR = 12.5%, FNR = 0.00%, OA = 98.62%, MagNA pure: SE = 91.47%, SP = 81.25%, PPV = 97.52%, NPV = 54.17%, FPR = 18.75%, FNR = 8.53%, OA = 90.34%). Comparison of AUC for AmpliLute method related to histological diagnosis was 0.935 (std. error 0.018, 95% CI (0.900–0.971), *P* < .001) and it was higher compared to AUC of MagNA pure method 0.877 (std. error 0.024, 95% CI (0.830–0.924), *P* < .001).

## 4. Discussion

Various research assays for HPV detection and typing have been used in epidemiological studies. The LA-HPV genotyping test provides a standardized, consistent and rapid means for HPV detection and genotyping. This test provides the capacity to identify 37 individual HPV genotypes within a given specimen and ascertain whether recurrent HPV positivity is, in fact, due to the persistence of a specific HR HPV genotype, meaning a substantially increased risk of disease progression [23–25]. Current specimen processing protocols recommend the use of manual extraction of DNA using the AmpliLute liquid media extraction kit, based on the QIAamp method (QIAGEN, Inc., Valencia, Calif, USA). This method of DNA preparation is time consuming and labor intensive and is prone to potential specimen cross-contamination, particularly when large numbers of specimens are being processed. An alternative method for DNA extraction is the automated MagNA pure LC extraction system, developed by the same company, which could facilitate the assay by minimizing the potential sample-contamination, hands-on time as well as increase labor efficiency and sample accuracy. 

 In the present study, we assessed DNA extracts form PreservCyt cervical samples, generated by the automated MagNA pure extraction system and by the manual AmpliLute method (both recommended by Roche) for HPV testing using the LA-HPV genotyping and detection assays. In addition, we correlated those results with the cytological and histological findings of the enrolled participants. 

Among the 253 ThinPrep Pap tests analyzed in our study, only one extract from the MagNA pure modified DNA-I extraction protocol was found to be invalid due to the absence of low and high *β*-globin. In contrast, all nucleic acids generated from the AmpliLute protocol were valid for HPV DNA genotyping. This marginal difference in sample adequacy could be either due to the high AmpliLute protocol efficiency or to the variations in aliquoting the specific sample resulting in an inadequacy of cellular material for the automated procedure. Comparison of the HPV genotyping results, obtained with the AmpliLute DNA to those from the MagNA demonstrated a substantial level of agreement (93.3%), with *κ* value of 0.864. Both extraction methods, in terms of qualitative results performed equally well when compared against the cytological diagnosis, with AmpliLute method demonstrating a small predominance (AUC of AmpliLute: 0.712 versus AUC of MagNA: 0.672). Nevertheless, the AmpliLute method exhibited higher sensitivity, specificity, positive and negative predictive values as opposed to MagNA method. The same outcome, AmpliLute method being more efficient than the MagNA, was noticed when we compared the two methods with the histological diagnosis. AUC of AmpliLute was 0.935 in contrast with the AUC of MagNA which was 0.877. 

HPV types identified in individual samples by each method are largely in agreement 90.1% (228/253). In all studied cases, AmpliLute showed a slightly higher detection rate of HPV compared with MagNA. For the former, HPV overall positivity was calculated at 57.3% comprising 30.4% of HPV detected as single infection and 27% as multiple. And for the latter, the respective positivity was 54.5%, 32% as single and 23% as multiple type of infection. The prevalence of HPV-infected samples increased, in both methods, with the severity of cytological diagnoses: 30.8% of AmpliLute versus 33% of MagNA in the WNL, 46% versus 44% of ASCUS, 84.6% versus 77% of LSIL, 100% versus 80% of LSIL/HSIL and 100% by both of HSIL. Since, there are limited Greek epidemiological data available, studies that yielded similar findings in healthy women to our results, were the report by Papachristou et al. [26] who found that the corresponding prevalence was 31.5% and Agorastos et al. [27] at 36.3%. Other studies in our country demonstrated that HPV DNA presence in WNL varied from 24% [28, 29] to 18% [30, 31] with the lowest prevalence reported at 2.5% [32]. This variability is also observed widely in the literature and is mainly due to the different criteria used for selecting the study population and also due to different molecular test applied. The biological meaning out of this is that latent HPV infections with no apparent underlying disease, which would otherwise not be diagnosed on cytological evaluation, are detectable with highly sensitive PCR-based methods. 

AmpliLute correctly identified 87.5% of the negative histological cases as HPV negative samples compared to 81.2% of MagNA. In addition, in cases with histological evaluation from HPV up to CIN2, MagNA missed 10 cases counting for 8% (10/123) of the population with these specific histological abnormalities, whereas all those cases were accurately detected as HPV positive samples by AmpliLute. In 144 cases with cytological findings of WNL and ASCUS, HPV was detected approximately in 52 cases (more than 50% of which were HR HPV types) and from which only 33 participants exhibited histological lesions of HPV up to CIN2. The remaining cases need to be followed up closely due to their elevated risk for developing a high-grade cervical lesion in the future. 

The small number of cases investigated in this present study limits our ability to conclude correct and representative epidemiological data on HPV prevalence in Greek women. Nevertheless, data of this report on HPV distribution add to a rich body on literature demonstrating that HPV 16 was the most frequent type detected in both types of infections followed by HR HPV 31, 53, 33, 45, 18, and 51. The observation of HPV 53 being among the three most prevalent HR HPV types detected is consistent with findings of previous Greek studies [28, 31]. However, the prevalence and clinical role of HPV 51 needs to be clarified through further studies. Critical points on multiple infections are succinctly presented, since the detailed analysis of multiple infections identified in the clinical specimens was beyond the scope of this work and they will be discussed analytically on other report. Nevertheless, they were highly detected among the HPV positive participants: 47% (68/145) by the AmpliLute and 42% (58/138) by the MagNA. Multiple type HPV infections were identified in approximately 50% of the HPV-infected individuals in WNL category, at 34% in ASCUS, at 50% in LSIL and in LSIL/HSIL and finally at 60% in HSIL category. The elevated incidence rate of multiple infections in our results are in line with the results described by Sandri et al. [10] who found multiple infections in 43% of the studied population and by Gargiulo et al. in 49.7% [33].

Regarding the discordant results observed between the two extraction methods, as showed in Table 3, in 44% (11/25) of the cases, MagNA failed in detecting HPV as opposed to the AmpliLute. Ten of these eleven cases were histological confirmed as ≥HPV and correctly identified by the AmpliLute. In 20% (5/25) of the cases, which were positive by the MagNA but negative by the AmpliLute method, there were either negative in histology or there were with normal cytology and without visible lesion upon colposcopy (thus for those women, cervical biopsy was not taken). In this regard, AmpliLute method gave a correct negative call and those cases could be considered as MagNA false positive results. In 32% (8/25) of cases, which were positive by the two methods but differing in the number of HPV genotypes detected, AmpliLute demonstrated higher level of detecting additional HPV genotypes in seven cases, apart from the common shared types, as opposed to MagNA. Those extra genotypes detected carry an increased clinical significance, since there were HR genotypes and could alter the clinical outcome of the patient. Only in one case, 4% (1/25) the HPV genotypes were completely different by the two methods and also in one case MagNA detected one extra genotype than the AmpliLute. The invalid result generated by the MagNA was HPV42 with CIN1 histology. Even though the patient population studied does not represent the general population attending our hospital, but only women who agreed to undergo further examination if necessary, the clinical samples tested covered a range of pathologies, from samples that were cytologically normal to samples that had HSIL. Therefore, the results (as well as the discordant result rate) for HPV detection generated by the two extraction methods demonstrated in the present study can be representative of the HPV infection in a screening population. 

It is important to mention that the decision of utilizing the modified DNA protocol for the automated MagNA pure extraction system, was made based on a recent report. It compares DNA extraction efficiencies using the same extraction system with the incorporation of three different working DNA extraction kits: (i) blood cells high-performance protol (DNA-I kit), (ii) total nucleic acid (TNA) kit, and (iii) a modified DNA-I kit with the manual AmpliLute protocol for both AMPLICOR and LA HPV tests in 150 specimens [34]. Although the women enrolled in the above study had histological confirmed cervical abnormalities, no comparison was made between the DNA extracts and the HPV genotyping test with their cytohistological findings. We used the modified DNA-I kit (blood cells high-performance protol) using 1 mL of PreservCyt sample as reported by Stevens et al. [34], since it performed better than the other two protocols and it was recommended by the author.

At this point, it is important to emphasise that even though for the manual AmpliLute method we used one fourth of biological material (250 *μ*L) as opposed to the automated MagNA DNA-I modified protocol (1000 *μ*L) and equal amount of DNA extract inputs were used for PCR amplification (50 *μ*L) and subsequently HPV detection, more HPV-positive cases were detected by the manual method. Someone would assume that increased HPV genotype detection would occur when a bigger amount of clinical sample is incorporated in the DNA extraction procedure, since more representative epithelial cells would be present in the sample tested, and thus increasing the possibility of HPV genotypes been detected by the assay used. The findings of our report, which are in contrast with previous reported one [34], declared the opposite, indicating that the current manual AmpliLute protocol for DNA preparation, provides adequate DNA quality, and consequently, it is capable of detecting HPV infections with high sensitivity. Having in mind that both methods gave comparative results when tested against cytology and histology, our data provide an additional advantage to AmpliLute, since reliable results can be obtained even when small volumes of biological material are available for molecular use. 

 In the literature, there is also a report that utilizing the same MagNA pure automated extraction system, compares the AMPLICOR HPV test to the INNO-LiPA HPV genotyping test, using only the TNA extraction kit for DNA isolation for AMPLICOR test [35], making, thus, difficult the direct comparison with this work. Several studies have undertaken assessment of the utility of various automated DNA extraction platforms in conjunction with the LA HPV test without comparing them with manual extraction methods [34, 36, 37]. Moreover, in the literature, there are limited studies that address the variability in HPV genotyping introduced by small changes in front-end DNA extraction procedures prior to use in the LA HPV genotyping test [38, 39]. From those reports, it was interesting found that minor changes to equally valid DNA extraction methods appeared to vary the assay's performance. For example, varying the volume of PreservCyt for DNA extraction or varying the centrifuge speed during DNA extraction or varying the amount of template DNA used for amplification can impact assay results. It is well documented and widely accepted that it is difficult to achieve reproducible and accurate HPV genotyping results using PCR-based methods, particularly when individual specimens may contain multiple concurrent infections and/or low viral copy numbers. Each of the many steps of testing, from collection of the cervical sample to the final recording of the result, can introduce important variability. Large-scale data comparing different methods of DNA isolation are needed to reach an optimal protocol for the HPV presence detection and accurate genotyping in order to monitor viral clearance, and most importantly HPV persistence, which is considered as a key factor in cervical cancer development. Moreover, accurate and sensitive methods for detection of HPV should be determined, since their performance can strongly affect the results of epidemiological studies and the clinical treatment strategy selected. Therefore, the MagNA extraction method should be tested against other automated and manual nucleic acids isolation techniques and in large population studies before being implemented and routinely used in laboratories. If would be proven accurate in detecting HPV infection, laboratories particularly those involved in large-scale HPV genotyping studies or handling a large amount of clinical specimens or can afford the cost of the automated procedure (more than two and a half times most pricey than the manual procedure) could profit from this automated nucleic acid isolation technique.

## 5. Conclusion

Accurate laboratory assays for the diagnosis of HPV infection are being recognized increasingly as essential for clinical management of women with cervical precancerous lesions. The first and most important step in molecular diagnosis of HPV infection is the nucleic acid isolation. An alternative approach to manual extraction procedures, which are time consuming and labor intense, is the automated processing of clinical specimens for HPV detection and genotyping which minimizes the potential sample contamination and the hands-on time. From our data, it was concluded that both DNA extraction methods demonstrated similar clinical performance, with no significant difference for any of the outcomes assessed even if for some outcomes the AmpliLute method exhibited higher sensitivity, specificity positive and negative predictive values as opposed to MagNA methods. Based on the results of this study, the automated nucleic acid isolation method should be tested versus other automated and manual techniques before it is routinely implemented. In addition, additional studies with larger populations are required to be carried out using the automated extraction system in order for its potential value to accurate HPV detection been determined.

## Figures and Tables

**Table 1:** Adequacy of 253 samples extracted by the two methods tested by HPV LA test.

Adequate sample	No. of samples (%)		
	Cytology	AmpliLute	MagNA pure*
Yes	247 (97,6%)	253 (100%)	252 (99,6%)
No	6 (2,4%)	0 (0,0%)	1 (0,4%)

Total	253	253	253

*DNA-I modified protocol using 1 mL PreservCyt sample.

**Table 2:** Concordance between AmpliLute and MagNA Pure (DNA-I, 1 mL)* DNA extracts, in single and multiple HPV infections, identified by LA assay.

No. of samples with the following LA results					
MagNA	AmpliLute				
	Single	Multiple (same)	Multiple (different)	Negative	Total
Single HPV type	73	5		2	80
Multiple HPV type (same)		52		3	55
Multiple HPV type (different)			3		3
Negative	4	7		103	114
Invalid	1				1

Total	78	64	3	108	253

**Table 3:** Analysis of cases with discrepant results.

Cytological findings	Histological findings	AmpliLute	MagNA Pure
#1 WNL	HPV	33, 67, 70	67, 70
#2 WNL	HPV	33, 45	(−)
#3 WNL	No biopsy	16, 31, 33, 45	(−)
#4 WNL	No biopsy	(−)	18
#5 WNL	Negative	33, 45	45
#6 WNL	No biopsy	(−)	6, 58
#7 WNL	No biopsy	(−)	16, 33, 35
#8 WNL	No biopsy	(−)	16, 62
#9 WNL	Negative	31, 33, 42	45
#10 ASCUS	HPV	35, 53	35, 53, 54
#11 ASCUS	HPV	39, 42, 52, 84	52
#12 ASCUS	HPV	6, 16, 52	52
#13 ASCUS	HPV	16	(−)
#14 LSIL	HPV	16	(−)
#15 LSIL	CIN 1	16, 31, 33, 45	16, 31
#16 LSIL	CIN 1	16, 31, 33, 45	(−)
#17 LSIL	CIN 1	42	Invalid
#18 LSIL	CIN 1	31, 16	(−)
#19 LSIL	Negative	(−)	59
#20 LSIL	HPV	16	(−)
#21 LSIL	CIN 1	6, 16, 33, 45	(−)
#22 LSIL	CIN 1	52, 82	52
#23 LSIL	CIN 1	16, 31	(−)
#24 LSIL	HPV	16	(−)
#25 LSIL/HSIL	CIN 2	16, 33, 51	(−)

**Table 4:** Distribution of HPV types (in single infections) detected by AmpliLute method against cytological diagnosis.

Cytology	AmpliLute																				
	HPV Negative	6	16	18	31	42	51	52	53	54	58	59	61	62	66	73	81	83	84	Multiple	Total
Negative	65	2	7		1				1			2	3			1		1		11	94
ASCUS	27		6	1	2			2	2					1						9	50
LSIL	14	4	14	1	3	4	1	1	2	1	2				1	1	1		2	39	91
LSIL/HSIL			2																	3	5
HSIL			1		1											1				4	7
Inadequate	2			1										1						2	6

Total	108	6	30	3	7	4	1	3	5	1	2	2	3	2	1	3	1	1	2	68	253

**Table 5:** Distribution of HPV types (in single infections) detected by MagNA pure method against cytological diagnosis.

Cyt ology	MagNA Pure																						
	HPV	6	16	18	31	42	45	51	52	53	54	58	59	61	62	66	73	81	83	84	Multiple	Inadequate	Total
	Negative																						
Negative	63	2	7	1	1		2			1			2	3			1		1		10		94
ASCUS	28		5	1	2				4	2					1						7		50
LSIL	20	4	11	2	3	3		1	2	2	1	2	1			1	1	1		2	33	1	91
LSIL/HSIL	1		2																		2		5
HSIL			1		1												1				4		7
Inadequate	2			1											1						2		6

Total	114	6	26	5	7	3	2	1	6	5	1	2	3	3	2	1	3	1	1	2	58	1	253

**Table 6:** Correlation of cytological findings to histological diagnosis.

Cytology	Histology						
	No biopsy	Negative	HPV	CIN 1	CIN 2	CIN 3	Total
WNL	79	2	4	9			94
ASCUS	27		14	8	1		50
LGIL		14	29	39	7	2	91
HGIL					4	3	7
LSIL/HSIL				1	3	1	5
Inadequate	2		4				6

Total	108	16	51	57	15	6	253

**Table 7:** Distribution of HPV types (in single infections) detected by AmpliLute method against Histological Diagnosis.

Hist ology	AmpliLute																				
	HPV6	16	18	31	42	51	52	53	54	58	59	61	62	66	73	81	83	84	Multiple	Negative	Total
Negative																			2	14	16
HPV	4	12	1		3	1	1	4	1	2			2	1	1	1		2	15		51
CIN 1		11	2	6	1		2												35		57
CIN 2		4		1											1				9		15
CIN 3		3																	3		6
No biopsy*	2							1			2	3			1		1		4	94	108

Total	6	30	3	7	4	1	3	5	1	2	2	3	2	1	3	1	1	2	68	108	253

*Women without colposcopic findings.

**Table 8:** Distribution of HPV types (in single infections) detected by MagNA Pure method against Histological Diagnosis.

His tology	MagNA pure																						
	HPV6	16	18	31	42	45	51	52	53	54	58	59	61	62	66	73	81	83	84	Inadequate	Multiple	Negative	Total
Negative						2						1										13	16
HPV	4	8	1		3		1	3	4	1	2			2	1	1	1		2		12	5	51
CIN 1		11	3	6				3												1	29	4	57
CIN 2		4		1												1					8	1	15
CIN 3		3																			3		6
No biopsy*	2		1						1			2	3			1		1			6	91	108

Total	6	26	5	7	3	2	1	6	5	1	2	3	3	2	1	3	1	1	2	1	58	114	253

*Women without colposcopic findings.
